# Metal (Pb, Cu, Cd, and Zn) Transfer along Food Chain and Health Risk Assessment through Raw Milk Consumption from Free-Range Cows

**DOI:** 10.3390/ijerph16214064

**Published:** 2019-10-23

**Authors:** Mirela Miclean, Oana Cadar, Erika Andrea Levei, Radu Roman, Alexandru Ozunu, Levente Levei

**Affiliations:** 1 Somes-Tisa Water Basin Administration, Cluj-Napoca 400213, Romania; mirelamiclean@yahoo.com; 2 INCDO-INOE 2000, Research Institute for Analytical Instrumentation, Cluj-Napoca 400293, Romania; oana.cadar@icia.ro (O.C.); erika.levei@icia.ro (E.A.L.); 3 Faculty of Animal Science and Biotechnologies, University of Agricultural Sciences and Veterinary Medicine, Cluj-Napoca 400372, Romania; raduroman94@gmail.com; 4 Faculty of Environmental Sciences and Engineering, Babes-Bolyai University, Cluj-Napoca 400294, Romania; alexandru.ozunu@ubbcluj.ro; 5 Disaster Management Training and Education Centre, University of the Free State, Bloemfontein 9300, South Africa

**Keywords:** human health risk assessment, metals, transfer factor, raw milk, food chain

## Abstract

Background: Metal transfer along the food chain has raised concerns about impacts on human health due to dietary exposure to low but chronic concentrations. Soil–forage–milk–consumer is a short food chain through which metals are able to reach an organism. Methods: Pb, Cu, Cd, and Zn were determined in water, soil, forage, and milk samples collected from free-range cattle farms situated near Baia Mare, Romania. The soil-to-forage (*TF_sf_*) and forage-to-milk (*TF_fm_*) transfer factors for metals and the health risk for three population groups (females, males, and children) through the consumption of milk containing low levels of metals were assessed. Results: *TF_sf_* indicated that the uptake capabilities of the metals from soil to forage were in the following order: Zn > Cd > Cu > Pb. *TF_fm_* indicated a lack of metal accumulation through forage ingestion. Estimated daily (*EDI*) and provisional tolerable weekly (*PTWI*) intake values revealed a minimal exposure of the population to those metals through milk consumption. A noncarcinogenic hazard index indicated that milk consumption from local markets does not pose any risk for human health; however, the average cancer risk showed a high potential carcinogenic risk. Conclusions: The consumption of milk produced by small local farmers does not pose noncarcinogenic risks. More extended studies should be carried out in order to identify the potential carcinogenic risk caused by the low levels of metals in the milk consumed.

## 1. Introduction

Animal-based food products such as dairy products play an important role in the human diet and have special significance in infant nutrition due to their protein, vitamin, and mineral contents [1,2,3]. Recently, there has been an increasing trend in the consumption of raw milk due to perceived health benefits compared to heat-treated milk, including increased nutritional value and the potential presence of probiotic bacteria [4]. Milk is a base element in the daily basket, and it has an important role especially in the diet of children; however, the data concerning the occurrence of essential and toxic elements in milk available in the Romanian market are scarce [3,5,6,7].

The “farm-to-fork” approach promoted by the European Union imposes control over key points in the food-producing chain, emphasizing primary production. In the case of food-producing animals, besides nutritional value, forage must be free of contaminants that could be transferred through the food chain until they reach humans. Forage quality is directly influenced by environmental quality and agronomic factors such as plant species, soil quality, fertilizing procedures, harvesting, processing, and storage [8].

The long and often uncontrolled use of metals (besides their inadequate disposal and prolonged persistence in the environment) and organisms has led to metal ubiquity in various environmental compartments. Furthermore, even at low concentrations, these contaminants are toxic to human health, so metal exposure has attracted considerable attention. As humans are at the top of the food chain, high contents of contaminants reach their organisms [9].

Soil–forage–milk–consumer is a short food chain through which metals are able to transfer to humans, causing health problems. In this specific case, soils are the primary source of metals that can be transferred to plants that become fodder for animals, and then they further pass through the digestive system of animals and are accumulated in lipid-rich tissues and partially excreted into milk. In addition, animals can ingest high amounts of soil during grazing [10].

The human health risk assessment (HHRA) is a methodology designed to estimate the probability of an adverse effect [11]. Human exposure to environmental contaminants could occur through the diet, with contaminant concentrations being the highest in animal-based food products (meat, milk, and dairy products). Long-term exposure to metals and their complete absorption through the digestive tract are assumed to be the worst-case scenario for the prediction of residual metal concentrations in animal-based food products. However, differences in animal physiology and growth rates should be considered [12]. Different methods are used to determine the ingested amount of food contaminants. Of these, the simplest deterministic model used to estimate chronic exposure combines food consumption data at the individual level with mean contamination data. In this regard, the contaminant concentrations are analyzed in food consumed by the population in areas chronically exposed to contaminants. To estimate metal intake through food, these results are associated with food consumption data [13].

The objectives of this study were to assess (i) long-term exposure to metals through milk consumption in the Baia Mare mining area by determining the mean intake through ingestion and its comparison to tolerable daily (*TDI*) and weekly (*TWI*) intakes; and (ii) the risk associated with raw milk consumption for local children and adults according to gender (male, female) using hazard quotients (*HQs*) for each studied metal and a hazard index (*HI*) to quantify the noncancer risk, carcinogenic risk (*CR*), and total carcinogenic risk (*TCR*). As cow milk is the most consumed milk type by the local inhabitants and is provided in the local markets as raw milk by local producers, the present study considered only small cow farms.

## 2. Materials and Methods

### 2.1. Sampling

**Figure 1:**
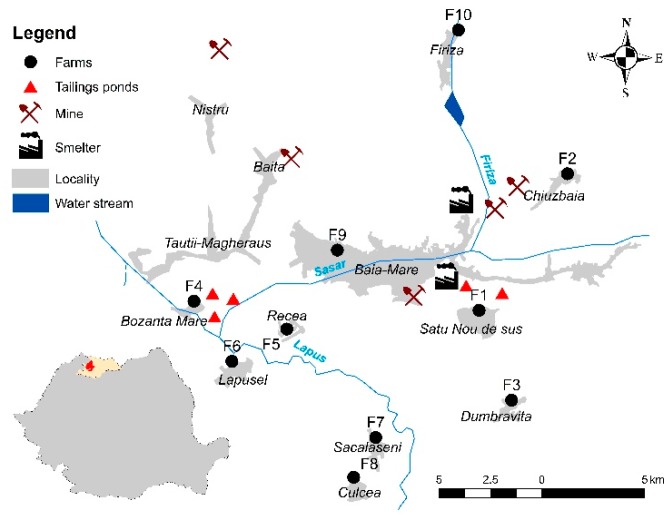
Study area.

Water, soil, forage, and milk samples were collected in May 2017 from 10 small cattle farms (about 5–25 cows from the Romanian Simmental and Holstein Friesian breeds) in a former mining area that is known for regularly delivering milk to the central market of the city of Baia Mare (north-western Romania) [11]. All mining and ore processing facilities were decommissioned in the last decade, and environmental reconstruction projects are being initiated. The farms (*F1–F10*) were situated in the vicinity of former nonferrous mining and ore processing facilities considered to be exposed to metal contamination (Figure 1). The water samples were collected from wells, ponds, or the drinking water network used for feeding the cattle. In order to have representative soil and forage samples, in each farm, 5 individual composite soil and forage samples were collected from 5 different points on the pasture. The soil samples were collected from 5 to 20 cm of depth using a stainless-steel shovel, while the aerial part of the forage was cut with a stainless-steel knife. The milk samples were collected in glass bottles and frozen at −20 °C until analysis during the morning milking.

### 2.2. Sample Preparation and Analysis

The water samples were passed through cellulose acetate membrane filters with a 0.45- µm pore size and stored at 4 °C until the analysis. The samples were acidulated to pH < 2 by adding 65% HNO_3_ (Merck) in accordance with Hoaghia et al. [14].

The soil samples were dried, ground, and sieved through a 2-mm sieve. In addition, 1 g of sample was digested in aqua regia (21 mL HCl 37% and 7 mL HNO_3_ 65%) for 16 h at room temperature and then 2 h in reflux conditions, filtered, and diluted to 100 mL with ultrapure water [15].

The freshly collected forage samples were washed using tap water, rinsed with distilled water, freeze-dried (FreeZone 2.5 Liter Benchtop freeze-dry system, Labconco, Kansas, MO, USA) at −40 °C and 25 psi for 3 days, and ground to obtain a homogenized powder. In addition, 1 g of sample was digested with 5 mL HNO_3_ 65% and 2 mL H_2_O_2_ 30% in closed polytetrafluoroethylene vessels using a MWS-3+ (Berghof, Eningen, Germany) microwave digestion system in accordance with the method described by Miclean et al. [11]. The digested samples were quantitatively transferred into 20-mL volumetric flasks and diluted to the mark with ultrapure water.

Volumes of 1 mL of the milk samples were microwave-digested with 8 mL HNO_3_ 65% and 2 mL H_2_O_2_ 30% according to the method described by Cadar et al. [3]. After mineralization, the digested samples were quantitatively transferred to 10-mL volumetric flasks and diluted to the mark with ultrapure water. The metal concentrations in milk were calculated considering a milk density equal to 1.0 g mL^−1^ [16].

The metal concentrations were measured using a PinAAcle 900T (Perkin-Elmer, Waltham, MA, USA) atomic absorption spectrometer (AAS) in flame mode (FAAS) for soil and furnace mode (GFAAS) for water, forage, and milk samples. The operating conditions were set according to Ivanova-Petropulos et al. [17]. For the quantification of the analytes, an external calibration was used with aqueous elemental standard solutions in ranges of 10–100 µg L^−1^ for Pb, 1.0–5.0 µg L^−1^ for Cd and Zn, and 5.0–25 µg L^−1^ for Cu, which was prepared from 1000 mg L^−1^ of monoelement solution (Merck, Darmstadt, Germany) with appropriate dilutions. The limit of detection (LOD) and limit of quantification (LOQ) were calculated as 3 times and 9 times the standard deviation of 10 blank measurements divided by the calibration curve slope. The measurement uncertainty was calculated through a bottom-up approach [18]. The accuracy of the metal determination was checked by analyzing certified reference materials (CRMs): “ERM-CC141 Loam Soil” (IRMM, Geel, Belgium), “SRM 1640a Trace Elements in Natural Water” (IRMM, Geel, Belgium), “IAEA-359 Cabbage” (IAEA, Vienna, Austria), and “NIST SRM-1549 Nonfat Milk Powder” (NIST, Gaithersburg, MD, US).

### 2.3. Transfer Factors

Metals can reach the human organism through a triple transfer: from soil to forage, from forage to milk, and from milk to humans. Transfer factors (*TFs*) quantify the transfer of metals between two consecutive levels of the food chain, namely soil–forage, forage–milk, and milk–human. The soil-to-forage transfer factors (*TF_sf_*), which indicate the metal uptake from soil by plants and show the human exposure to metals through the food chain, were calculated according to Equation (1) [19]. The forage-to-milk transfer factors (*TF_fm_*) were calculated according to Equation (2) [20].

In this study, the following assumptions were made: (i) the cows were exposed to forage with relatively constant metal contents for long periods of time; (ii) the cows’ feeding was based mainly on grazing from the study area; and (iii) the absorption and excretion rates were proportional to the contaminant concentration. Equation (1) is

(1) $$TF_{sf}=\frac{C_{f}}{C_{s}},$$

where *C_f_* is the metal content in forage (mg kg^−1^ dw (dw: dry weight)), and *C_s_* is the metal content in soil (mg·kg^−1^ dw). Equation (2) is

(2) $$TF_{fm}=\frac{C_{m}}{C_{f}},$$

where *C_m_* is the metal content in milk (mg·kg^−1^ ww (ww: wet weight)), and *C_f_* is the metal content in forage (mg·kg^−1^ dw).

### 2.4. Health Risk Assessment

To assess the potential health risk for humans associated with milk consumption, dietary exposure was determined by calculating the estimated daily intake (*EDI*) for humans based on milk consumption frequency questionnaires (Equation (3)):

(3) $$EDI=C_{m}\times F_{IR},$$

where *EDI* is the estimated daily intake (µg·day^−1^), *Cm* is the average concentration of metal in milk (µg·g^−1^, ww), and *FIR* is the milk ingestion rate (g·day^−1^) determined by frequency questionnaires filled out by 75 persons, namely 55 adults (28 male and 27 female) and 20 children (200 g·day^−1^ for females, 300 g·day^−1^ for males, and 500 g·day^−1^ for children). *EDI* is a value related to the metal concentration in milk and the daily consumption of milk and body weight (bw), which influences tolerance to contaminants [9].

The estimated weakly intake (*EWI*), which is calculated by multiplying *EDI* by 7, was compared to the provisional tolerable weekly intake (*PTWI*) [21,22,23]. The tolerable daily intake limits of Pb, Cd, Zn, and Cu for adults are 200, 57–71, 33,000, and 6500 μg·day^−1^, respectively [24], while the provisional tolerable weekly intake (*PTWI* in μg·bw^−1^·week^−1^) is 25 for Pb and 7 for Cd [25]. The *PTWI* values expressed as μg·week^−1^ were calculated by multiplying *PTWI* values expressed as µg·bw^−1^·week^−1^ by bw (75 kg for males, 65 kg for females, and 30 kg for children), which resulted in the following values: 1625 μg·week^−1^ (females), 1875 μg·week^−1^ (males), and 750 μg·week^−1^ (children) for Pb and 1625 μg·week^−1^ (females), 1875 μg·week^−1^ (males), and 750 μg·week^−1^ (children) for Cd.

The noncarcinogenic health risk through milk consumption was quantified using hazard quotients (*HQs*) for each investigated metal according to Equation (4) [26]:

(4) $$HQ=\frac{EF\times ED\times F_{IR}\times C}{RfD\times W\times T_{A}}\times 10^{-3},$$

where *EF* is the exposure frequency (350 days year^−1^), *ED* is the exposure duration (52 years for adults and 18 years for children) equivalent to an average lifetime when consuming milk, *F_IR_* is the food ingestion rate considered (300 for males, 200 for females, and 500 for children (g day^−1^)), *C* is the metal concentration in milk (mg kg^−1^), *RfD* is the oral reference dose (considered to be Pb 0.004, Cd 0.001, Cu 0.04, and Zn 0.30 mg kg^−1^·day^−1^), *W* is the mean body weight (75 kg for males, 65 kg for females, 30 kg for children), and *T_A_* is the mean exposure time to noncarcinogenic metals [27]. Due to the fact that the investigated residents usually consume raw milk, the ingested dose was considered to be equal to the concentration of metals in milk [24].

An *HQ* value less than 1 indicates that the daily exposure to a certain metal through milk consumption is unlikely to cause noncarcinogenic health effects, while an *HQ* value greater than 1 shows that in the exposed population, chronic health risks may occur [28].

The health risk through simultaneous exposure to different metals was expressed by a hazard index (*HI*) calculated as the sum of the hazard quotients of each metal. A health risk assessment through the calculation of an *HI* is based on the assumption that simultaneous exposure to two or more contaminants could have additive and/or interactive effects [25]. In the case of HI < 1, milk consumption is safe, while in the case of *HI* > 1, milk consumption may pose a health risk.

The probability that an individual develops cancer over a lifetime of exposure to contaminated milk consumption was estimated using carcinogenic risk (*CR*). The *CR* was calculated for Pb and Cd according to Equation (5). The total carcinogenic risk (*TCR*) was calculated as the sum of the *CR* for Cd and for Pb, assuming their additive effects. A *CR* of 10^−6^, which indicates a probability of 1 in 1,000,000 individuals, was considered acceptable [29]:

(5) $$CR=\frac{C\times E_{F}\times E_{D}\times F_{IR}\times 10^{-3}}{T_{A}\times W}\times CSF$$

where *C*, *EF*, *ED*, *F_IR_*, *W*, and *T_A_* are similar to Equation (4), and *CSF* is the carcinogenic slope factor, which estimates the upper-bound probability of an individual developing cancer as a result of a lifetime of exposure to a particular level of a potential carcinogen through an ingestion route (Cd 15 and Pb 0.0085 mg kg^−1^ day^−1^) [27,30].

## 3. Results and Discussion

### 3.1. Quality Assurance Data

The analytical method performance is presented in Table 1. Mean recoveries for the determination of metals in CRMs ranged between 87% and 114%. The performance parameters show that the used methods were suitable for application to routine methods for the risk assessment of investigated metals in milk.

### 3.2. Metal Contents in Soil, Water, Forage, and Milk Samples

Cu, Pb, Zn, and Cd were detected in all soil, water, forage, and milk samples (Table 2), suggesting possible pollution with these elements. The mean metal content decreased in the order of Zn > Pb > Cu > Cd in the case of soil and Zn > Cu > Pb > Cd in the case of water, forage, and milk. The alert threshold was exceeded in more than two-thirds of the soil samples for Pb and Cd and one-third of the soil samples for Cu and Zn, while the intervention threshold was exceeded in about half of the samples for Pb and Cd and in one sample for Cu. Generally, a high variability of all metal contents was observed, probably as a consequence of the presence of legacy pollution sources related to mining and ore processing activities, especially poorly managed mine tailings that favor the dispersion of metal-rich dust in the surrounding area. These data confirmed that soil is the main metal pollution vector of vegetation, which is forwarded to food. Similar levels of metals in soil in the Baia Mare area were found earlier by Miclean et al. [15] and Levei et al. [31]. In the Baia Mare area, the metal mobility is highly variable: 1.3–80% of Cd, 2.2–40% of Pb, 2.0–34% of Cu, and 0.3–21% of Zn content are considered to be bioavailable [31].

Generally, animals receive metals mainly through water and food. In the study area, free-range cattle are pasture-fed, grazing from spring to autumn and consuming forage composed mainly of dried forage during winter.

In our case, the Cd, Cu, and Zn contents in water were below (and the Pb content exceeded in one sample) the corresponding maximum admitted concentration. Thus, the metal intake from water was considered negligible compared to other dietary metal sources. Forage is another important metal-transporting vector, as it influences the quality of food products resulting from animals that are fed with forage. However, in our case, the legislated thresholds for Pb and Cd were not exceeded, except for Cd in one sample. There are no set thresholds for Cu and Zn, as their role in an organism can be beneficial or toxic depending on their concentration. The average levels of Pb and Cd in forage from the Baia Mare area were lower than those reported by Caggiano et al. [36] in forage used for ovine in southern Italy.

Lead is a toxic and possible carcinogenic element. Despite the fact that the metal content was low in water and moderate in forage, the legislated maximum level for Pb in milk was exceeded in half of the samples, indicating that the milk was not suitable for human consumption. A possible explanation could be the existence of other metal intake sources besides forage and water. Two such sources that were identified were the ingestion of contaminated soil (adhered to vegetation) through grazing and the mixing of soil with fodder during fodder preparation. Another source could have been the trace element premixes and supplementary minerals introduced into cows’ diets. Moreover, the Pb content in dairy products could have been a consequence of casein’s chemical affinity for Pb [37]. Cd is another toxic and carcinogenic element that can reach the human organism through food intake. Cu and Zn are essential elements; nevertheless, in excess they may result in adverse effects. On the basis of their content in milk, the levels of Cu, Zn, and Cd do not represent a toxicological risk in the Baia Mare area. However, as metal intake is accumulative, an analysis of the entire diet of the population in this area is needed for assessing the global metal toxicological risk [38]. The mean values of the metals were comparable to those obtained in raw and pasteurized milk collected in the Cluj-Napoca area [6] and higher than those from the Rodnei Mountains [3]. The Zn and Cu contents in milk were lower, while the Cd and Pb contents were comparable to those reported in raw cow milk from Transylvanian farms in Romania [5]. The Pb values were generally higher than those reported in raw cow milk from dairy farms close to mines in Gauteng and the northwest provinces of South Africa [39] and in rural areas of Croatia [40]. Compared to the metal levels in cow milk obtained from supervised and unsupervised feeding mainly with corn, oats, and alfalfa grown in wastewater-irrigated areas in the Mezquital Valley, Mexico City, the metal contents obtained in our study were lower for Zn and Pb and comparable in the case of Cu [38]. The average content of Pb in milk was comparable, while the content of Cd was higher than in raw cow milk from China [41]. The median values for Cu, Cd, and Pb were higher (and those of Zn were comparable to) those reported by Rodriguez-Bermudez [42] in raw cow milk from organic and conventional farms in northern Spain.

### 3.3. Transfer Factors from Soil to Forage and from Forage to Milk

The soil-to-plant transfer factor (also called the uptake factor, accumulation factor, or concentration factor) is an index used to evaluate the transfer potential of a metal from soil to plants. Table 3 summarizes the descriptive statistical results for the *TF_sf_* of metals from soil to forage, which was calculated as the ratio of metal concentrations in forage to those in the corresponding soil. In all cases, the TFs were below 1, which indicates that the concentrations of metals in soil were higher than in forage and that this was the only source of contamination in forage, regardless of the absorption of metals from air deposition or other unknown sources. The values of *TFsf* decreased in the following order: Zn > Cd > Cu > Pb.

The *TFs* of metals from soil to forage varied between 0.005 (Pb) and 0.208 (Zn), which was two orders of magnitude. The transfers of Cd and Pb were the most threatening due to the high toxicity of both metals. The average *TF_sf_* values for Cd, Cu, and Zn were significantly lower than for Pb, showing that these metals are much easier transferred from soil to forage. Cd, Cu, and Zn have similar ionic radii and the same way of disturbing nucleic acid metabolism. Once Cd enters into the cell of a plant, it combines with enzymes instead of Zn, resulting in an easier transfer from soil to the edible part of a vegetable (for Cd compared to Zn). The relatively lower *TF_sf_* values for Pb indicate that it is much more difficult for Pb to transfer from soil to forage [24]. These results were in accordance with those of Zhou et al. [43], who reported that Pb in soil is not the main source of the edible part of a vegetable, especially leafy vegetables.

Kloke et al. [44] reported generalized *TFs* for soils and plants based on the root uptake of metals: Cu and Pb (0.01–0.1) and Cd and Zn (1–10). These TFs were based on metal absorption through roots and plant surfaces and subsequent atmospheric deposition [44]. It can be concluded that even when the types of forage are different, the order of metal-absorbing capability from soil to forage is similar. Our results were in good agreement with these results, indicating high *TF_sf_* values for Cd, Pb, and Zn compared to Cu due to the high mobility and phytoavailability of these metals, which is a reflection of their relatively poor sorption in soils. Thornton et al. [45] considered that a plant/soil ratio of 0.1 for any metal indicates that the plant is excluding the metal from its tissues. When the metal concentrations in soil are high, only a part of root uptake is transferred to the leaves, giving a leaf/soil concentration ratio of about 0.2. Therefore, a transfer factor above 0.2 could indicate the anthropogenic contamination of plants.

Table 3 also summarizes the descriptive statistical results of the *TFs* of metals from forage to milk, which were calculated as the ratio of the metal concentrations in milk to those in the corresponding forage. In all cases, *TF_fm_* was below 1, which indicates that the concentrations of metals in forage were higher than in milk and that there was a lack of metal accumulation through vegetation (forage) ingestion [46]. Metal *TFs* from forage to milk varied between 0.006 (Cd) and 0.247 (Zn), which was two orders of magnitude, in the following order: Zn > Cu > Pb > Cd.

The following limitations should be considered: the *TFs* of smaller animals are higher than for larger animals, and those of adults are lower than those of young livestock. These differences are because *TFs* incorporate dry matter intake, which increase with animal size. An alternative method of quantifying the transfer from forage to milk could be the equilibrium ratio of the metal concentration in milk (ww) to that in forage (dw) [47].

### 3.4. Health Risk Assessment through Milk Consumption

The degree of toxicity of metals to human beings depends upon their daily intake rate. In this framework, the international health authorities have set permissible maximum tolerable intakes for both toxic metals and essential elements (for those that produce undesirable effects) [21,22,23].

Regarding the daily intake of metals through milk ingestion for the investigated residents, for each investigated metal, the mean dietary exposure varied in the following order: *EDI_children_* > *EDI_male_* > *EDI_female_* (according to daily milk consumption by each population group) (Table 4).

Possible factors contributing to the *EDI* of metals are the quantity of consumed milk and mean bw. For the investigated metals, the *EDI* values varied as follows: Zn > Cu > Pb > Cd, with a maximum dietary exposure of 2192 µg Zn person^−1^ day^−1^ for children and a minimum of 0.558 µg Cd·person^−1^·day^−1^ for females. Christophoridis et al. [25] also reported low heavy metal (Cd, Pb, and Hg) intake in the average consumption of cheese products in Greece, while Harmanescu et al. [48] revealed that the *EDI* of metal rates was higher in females compared to males (through vegetable consumption).

The mean daily and weekly intakes of Pb and Cd, respectively, through milk consumption of the investigated residents (exposed to chronic pollution) were lower than the reference values for *TDI* and *PTWI*, respectively, indicating that milk consumption does not pose a health risk for residents in the studied area. However, the *TDI* values recommended by the World Health Organization (WHO) refer to total metal intake through all absorption paths: ingestion, inhalation, and dermal contact [49]. Thus, the *TDI* values could be exceeded, especially for children, indicating a potential health risk. 

The hazard quotients (*HQs*) for Cu, Pb, Zn, and Cd for milk consumption in the studied area for males, females, and children are shown in Table 5.

The obtained *HQ* values were lower than 1, indicating no potential health risk through milk consumption, in the investigated area. The mean hazard quotients calculated for milk consumption decreased in the following order: *HQ_Zn_* > *HQ_Cd_* ≥ *HQ_Cu_* ≥ *HQ_Pb_* for all studied population groups. The *HQ* values for males and females did not differ significantly for any of the studied metals, but were much higher for children. The *HQ* values in our study for raw milk consumption were much higher than those reported for the population of Hamadan City in western Iran [50].

The calculated noncarcinogenic hazard index (*HI*) was lower than 1 for all studied population groups, indicating that milk consumption from local markets does not pose any risk for human health. 

**Figure 2:**
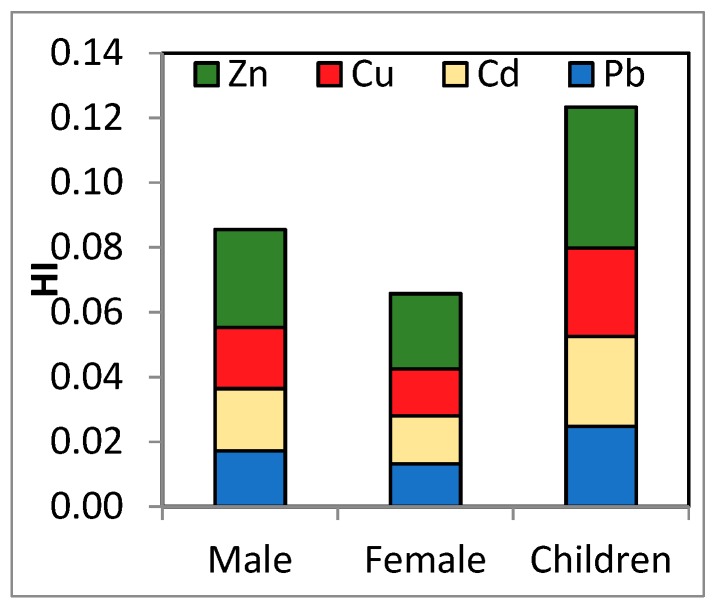
Contribution of *HQs* to the *HI* by population group.

The element that made the greatest contribution to *HI* in the majority of samples was Zn (Figure 2). No differences between the risk for males and females were observed. However, the average *HI* was about two times higher for children than for adults, suggesting that if more types of contaminated food categories are consumed, the risk may increase significantly. This fact is possible especially if milk consumption is associated with the consumption of vegetables grown in contaminated soils. Thus, if we took into consideration all contamination through dietary exposure, the exposure to these metals might be higher. Children are especially susceptible to developing noncarcinogenic health effects. As their undeveloped digestive tracts favor the absorption of toxic metals [38]. A much lower *HI* was reported both for adults and children after milk consumption in Iran [50].

The average *CR* values (Table 6) for Pb-contaminated milk were below (while for Cd-contaminated milk they were above) the lowest acceptable risk values, indicating a high potential carcinogenic risk from milk consumption in the Baia Mare area. The average carcinogenic risk of Pb was below 10^−6^ for adults and slightly above that for children, but this was negligible compared to that of Cd. Cd was found to be the highest contributor to total cancer risk for all population groups. The total cancer risk for males and females was comparable, but it was slightly higher for children.

Although metal intake through milk is an important exposure pathway, exposure to metals may also occur through the ingestion of other food categories or through other pathways such as inhalation or dermal contact [51]. Thus, our data underestimate the real noncarcinogenic and carcinogenic risks in the study area. However, the great ability of the human organism to cope with extreme negative conditions through various mechanisms such as excretion or limited intestinal absorption can limit the negative health effects of toxic metals. Despite all this, special attention to food quality is strongly recommended, especially for children, not only in polluted areas but worldwide. 

## 4. Conclusions

The tendency of metals to accumulate in tissues, their persistence, and the high health risk have raised concerns about the impact on human health due to dietary exposure to low but chronic concentrations. The consumers’ health risk assessment in the area investigated was based on estimated daily and weekly intakes, hazard coefficients, and a carcinogenic risk index. The study was conducted in 10 small cattle farms in the Baia Mare area, north-western Romania, and showed low levels of Cu, Zn, and Cd and high levels of Pb in milk consumed by locals. For each investigated metal, the estimated average dietary exposure varied in the order of *EDI*_children_ > *EDI*_male_ > *EDI*_female_, which is consistent with daily milk consumption. The average daily and weekly Pb and Cd intake through milk for the investigated residents exposed to chronic pollution was lower than the *TDI* and *PTWI* reference values, indicating that milk intake does not cause a health risk for the inhabitants of the area. However, taking into consideration that WHO-recommended *TDI* values refer to the total intake of metal in the body through all penetration pathways, these baseline values may be exceeded, especially in the case of children, indicating a potential risk to their state of health. The individual average target hazard ratios calculated for milk consumption in the studied area decreased in the order of *THQ_Zn_* > *THQ_Cd_* > *THQ_Cu_* > *THQ_Pb_* for each investigated population group and decreased in the order of *THQ_children_* > *THQ_male_* > *THQ_female_* for each studied metal. The outcome of the risk assessment indicated that the inhabitants of the investigated area do not experience noncarcinogenic health risk, but carcinogenic risk could appear in the case of long-term exposure to Cd through milk.

## Figures and Tables

**Table 1:** Analytical method performance parameters: limit of detection (LOD), limit of quantification (LOQ), and expanded uncertainty (U).

Analyte	Matrix	LOD	LOQ	U (%)
Pb	Soil (mg kg^−1^)	0.33	1.00	9.2
	Water (mg L^−1^)	0.30	0.90	7.5
	Forage (mg kg^−1^)	0.006	0.018	9.0
	Milk (mg kg^−1^)	0.003	0.009	9.8
Cd	Soil (mg kg^−1^)	0.11	0.33	11.2
	Water (mg L^−1^)	0.10	0.30	6.4
	Forage (mg kg^−1^)	0.002	0.006	9.6
	Milk (mg kg^−1^)	0.001	0.003	10.1
Cu	Soil (mg kg^−1^)	0.22	0.66	8.7
	Water (mg L^−1^)	0.20	0.60	6.3
	Forage (mg kg^−1^)	0.004	0.012	10.1
	Milk (mg kg^−1^)	0.002	0.006	10.5
Zn	Soil (mg kg^−1^)	0.11	0.33	10.3
	Water (mg L^−1^)	0.33	1.00	3.2
	Forage (mg kg^−1^)	0.007	0.020	9.3
	Milk (mg kg^−1^)	0.003	0.010	9.9

**Table 2:** Metal content in soil (mg kg^−1^ dw (dw: dry weight)), water (µg L^−1^), forage (mg kg^−1^ dw), and milk (mg kg^−1^ ww (ww: wet weight)) in the Baia Mare area and the legislated limit values (LVs) and % of samples exceeding the LVs.

Concentration (*n* = 10)		Pb	Cd	Cu	Zn
Soil	Range	12.4–479	0.75–8.36	38.3–211	117–590
	Mean ± SD	205 ± 164	4.34 ± 2.54	95.9 ± 55.2	283 ± 160
	Median	187	3.78	77.3	220
	LV ^1^	50/100	3/5	100/200	300/600
	*n* > LV	70%/60%	60%/40%	30%/10%	30%/0%
Water	Range	2.70–11.7	0.340–2.80	24.8–85.2	673–1570
	Mean ± SD	5.20 ± 2.51	0.816 ± 0.761	51.7 ± 19.5	1041 ± 320
	Median	4.82	0.535	49.9	891
	LV ^2^	10	5	100	5000
	*n* > LV	10%	0%	0%	0%
Forage	Range	0.15–2.24	0.10–1.44	3.43–10.8	13.4–53.9
	Mean ± SD	0.80 ± 0.66	0.41 ± 0.42	6.37 ± 2.20	28.4 ± 13.8
	Median	0.59	0.26	6.33	28.9
	LV ^3^	30	1.0	-	-
	*n* > LV	0%	10%	-	-
Milk	Range	0.010–0.048	0.003–0.011	0.095–0.446	2.38–4.38
	Mean ± SD	0.024 ± 0.015	0.007 ± 0.003	0.265 ± 0.111	3.18 ± 0.665
	Median	0.018	0.007	0.286	2.94
	LV ^4^	0.02	-	-	-
	*n* > LV	50%	-	-	-

^1^ Alert/intervention level for sensitive soil use according to Order 756/1997 [32]; ^2^ maximum admitted concentration according to Law 311/2004 [33]; ^3^ Directive 2002/32/EC [34]; ^4^ Commission Regulation (EC) 1881/2006 [35].

**Table 3:** Descriptive statistics of soil-to-forage (*TF_sf_*) and forage-to-milk (*TF_fm_*) metal transfer factors.

		Pb	Cd	Cu	Zn
*TF_sf_*	Range	0.002–0.012	0.035–0.187	0.039–0.122	0.068–0.208
	Mean ± SD	0.005 ± 0.003	0.094 ± 0.057	0.076 ± 0.025	0.107 ± 0.041
	Median	0.005	0.060	0.070	0.092
*TF_fm_*	Range	0.015–0.075	0.006–0.067	0.022–0.071	0.075–0.247
	Mean ± SD	0.040 ± 0.021	0.028 ± 0.021	0.042 ± 0.015	0.133 ± 0.061
	Median	0.037	0.021	0.040	0.117

**Table 4:** Mean estimated daily intake (*EDI*) and estimated weekly intake (*EWI*) of metals.

Metal	*EDI*, µg·day^−1^			*EWI*, µg·week^−1^		
	Female	Male	Children	Female	Male	Children
Pb	4.82	7.24	12.1	33.8	50.6	84.4
Cd	1.35	2.03	3.38	9.45	14.2	23.6
Cu	53.0	79.5	132	371	556	927
Zn	636	953	1589	4449	6674	11123

**Table 5:** Hazard quotients (HQs, *n* = 10) and hazard index (*HI*) for Pb, Cd, Cu, and Zn intake through milk consumption in the Baia Mare area.

*HQ/HI*	*n* = 10	Male	Female	Children
*HQ_Pb_*	Range	0.007–0.034	0.005–0.026	0.010–0.049
	Mean ± SD	0.017 ± 0.011	0.013 ± 0.008	0.025 ± 0.015
	Median	0.013	0.010	0.019
*HQ_Cd_*	Range	0.008–0.032	0.006–0.025	0.011–0.047
	Mean ± SD	0.019 ± 0.009	0.015 ± 0.007	0.028 ± 0.013
	Median	0.019	0.014	0.027
*HQ_Cu_*	Range	0.007–0.032	0.005–0.024	0.010–0.046
	Mean ± SD	0.019 ± 0.008	0.015 ± 0.006	0.027 ± 0.011
	Median	0.020	0.016	0.029
*HQ_Zn_*	Range	0.023–0.042	0.017–0.032	0.033–0.060
	Mean ± SD	0.030 ± 0.006	0.023 ± 0.005	0.044 ± 0.009
	Median	0.028	0.021	0.040
*HI*	Range	0.050–0.131	0.038–0.101	0.072–0.189
	Mean ± SD	0.085 ± 0.030	0.066 ± 0.023	0.123 ± 0.044
	Median	0.080	0.062	0.116

**Table 6:** Carcinogenic risk (*CR*, *n* = 10) and total carcinogenic risk (*TCR*) from Pb and Cd intake through milk in the Baia Mare area.

*CR/TCR*	*n* = 10	Male	Female	Children
*CR_Pb_*	Range	2.4 10^−7^–1.2 10^−6^	1.8 10^−7^–9.0 10^−7^	3.4 10^−6^–1.7 10^−5^
	Mean ± SD	5.8 10^−7^ ± 3.6 10^−7^	4.5 10^−7^ ± 2.8 10^−7^	8.4 10^−6^ ± 5.2 10^−6^
	Median	4.4 10^−7^	3.4 10^−7^	6.4 10^−6^
*CR_Cd_*	Range	1.2 10^−4^–4.9 10^−4^	9.2 10^−5^–3.7 10^−4^	1.7 10^−4^–7.0 10^−4^
	Mean ± SD	2.9 10^−4^ ± 1.4 10^−4^	2.2 10^−4^ ± 1.0 10^−4^	4.2 10^−4^ ± 2.0 10^−4^
	Median	2.8 10^−4^	2.2 10^−4^	4.1 10^−4^
*TCR*	Range	1.2 10^−4^–4.9 10^−4^	9.2 10^−5^–3.8 10^−4^	1.8 10^−5^–7.2 10^−4^
	Mean ± SD	2.9 10^−4^ ± 1.4 10^−4^	2.2 10^−4^ ± 1.0 10^−4^	4.2 10^−4^ ± 2.0 10^−4^
	Median	2.8 10^−4^	2.2 10^−4^	4.1 10^−4^
